# Decreased prevalence and severity of asthma symptoms among adolescents in Ibadan, Nigeria, 1995–2018

**DOI:** 10.5588/ijtld.23.0138

**Published:** 2023-12-01

**Authors:** A. A. Labaeka, A. G. Falade, E. O. D. Addo-Yobo, K. Mortimer, L. Zurba, M. Lesosky, P. Ellwood, M. I. Asher

**Affiliations:** 1 Department of Paediatrics, College of Medicine, University of Ibadan, Ibadan,; 2 Department of Paediatrics, University College Hospital, Ibadan, Nigeria;; 3 Kwame Nkrumah University of Science and Technology, Kumasi,; 4 Komfo Anokye Teaching Hospital, Kumasi, Ghana;; 5 Liverpool University Hospitals NHS Foundation Trust, Liverpool, UK;; 6 University of KwaZulu Natal, Durban,; 7 Spirometry Training Services Africa, Durban,; 8 Division of Epidemiology & Biostatistics, School of Public Health, University of Cape Town, Cape Town, South Africa;; 9 Department of Paediatrics, Child and Youth Health, University of Auckland, Auckland, New Zealand

**Keywords:** asthma severity, asthma prevalence, ISAAC, Global Asthma Network, GAN

## Abstract

**BACKGROUND:**

Phases I and III of the International Study of Asthma and Allergies in Childhood (ISAAC) documented increased asthma symptoms among Nigerian 13–14-year old adolescents. We investigated the trend further using the Global Asthma Network (GAN) surveillance.

**METHODS:**

Using ISAAC methodology, GAN Phase I data on symptoms and risk factors for asthma and asthma management were obtained from February to July 2018.

**RESULTS:**

There were 2,897 adolescents from 23 secondary schools. For current wheeze, there was an absolute prevalence fall per decade of –1.4 with –1 standard error (SE) in 16 years from 2002 (ISAAC Phase III) to 2018 (GAN Phase I). This pattern was evident for prevalence of reported asthma ever, severe asthma symptoms and night cough with ≥1 SE. During the 23-year interval between ISAAC Phase I and GAN Phase I, there was a fall (≥1 SE) in the absolute prevalence of reported asthma ever, severe asthma symptoms and night cough, except for severe asthma symptoms (–0.2 SE). Respectively 36% and 43% of symptomatic adolescents purchased and used salbutamol and prednisolone.

**CONCLUSION:**

The prevalence and severity of asthma symptoms remain high among adolescents in Ibadan. This could be mitigated by improved access to affordable and effective asthma treatments.

## Abstract (fr)

**CONTEXTE:**

Les Phases I et III de l’étude internationale de l’asthme et des allergies dans l’enfance (ISAAC, pour l’anglais *International Study of Asthma and Allergies in Childhood*) ont mis en évidence une augmentation des symptômes d’asthme chez les adolescents nigérians de 13 et 14 ans. Nous avons étudié cette tendance de manière plus approfondie en utilisant la surveillance du Réseau mondial de l’asthme (GAN, pour l’anglais, *Global Asthma Network*).

**MÉTHODES:**

En utilisant la méthodologie ISAAC, les données de la Phase I du GAN sur les symptômes et les facteurs de risque de l’asthme et la prise en charge de l’asthme ont été obtenues de février à juillet 2018.

**RÉSULTATS:**

Il y avait 2 897 adolescents provenant de 23 écoles secondaires. Pour la respiration sifflante actuelle, il y avait une baisse de prévalence absolue par décennie de –1,4 avec –1 erreur standard (SE) en 16 ans de 2002 (ISAAC Phase III) à 2018 (GAN Phase I). Cette tendance était évidente pour la prévalence de l’asthme déclaré, des symptômes d’asthme graves et de la toux nocturne avec ≥1 SE. Au cours de l’intervalle de 23 ans entre ISAAC Phase I et GAN Phase I, il y a eu une baisse (≥1 SE) de la prévalence absolue de l’asthme déclaré, des symptômes d’asthme sévère et de la toux nocturne, sauf pour les symptômes d’asthme sévère (–0,2 SE). Respectivement 36% et 43% des adolescents symptomatiques ont acheté et utilisé du salbutamol et de la prednisolone.

**CONCLUSION:**

La prévalence et la sévérité des symptômes de l’asthme restent élevées chez les adolescents d’Ibadan. Ceci pourrait être atténué par un meilleur accès à des traitements abordables et efficaces de l’asthme.

Asthma is the most common chronic childhood disease.^1^,^2^ Global data on asthma prevalence and severity, which can be effectively compared across different populations, dates back over a decade, and was last undertaken by the International Study of Asthma and Allergies in Childhood (ISAAC)^3^,^4^ that studied two ages of school pupils (6/7-year-old children and 13/14-year-old adolescents). In two cross-sectional studies, ISAAC found a marked increase of asthma prevalence in Ibadan, Nigeria.^3^ In line with Sustainable Development Goal (SDG) 3,^4^,^5^ which aims to reduce premature mortality from non-communicable diseases by one-third through prevention and treatment, it is imperative to ensure the availability of affordable, quality-assured asthma medications and medical care to all individuals. However, essential asthma medications (inhaled salbutamol and corticosteroids) are largely not available and mostly unaffordable in many low- and middle-income countries (LMICs), including Nigeria.^6^,^7^ Inhalable corticosteroids (ICS) and ICS-long-acting beta-agonist (LABA) were found in respectively 15.6% and 47.7% of pharmacies in Nigeria. In contrast, the majority of pharmacies had oral corticosteroids (72.7%) and oral salbutamol (71.1%).^8^

Ibadan took part in ISAAC Phases I (1995) and III (2001/2002), involving adolescents aged 13–14 years in secondary school settings; a significant increase from 10.7% to 13.0% was found in the prevalence of wheeze over the 6-year period.^9^

Building on the ISAAC approach, the Global Asthma Network (GAN) was founded in 2012. The recently completed GAN Phase I cross-sectional study focuses on global surveillance of prevalence, severity, management and risk factors for asthma.^10^–^12^ This GAN Phase I study of the prevalence and severity of asthma among adolescents in Ibadan enables comparisons with previous ISAAC findings and the medical care accessible to them.

GAN Phase I in Nigeria was undertaken to address the following issues: 1) the changing burden of asthma in Nigerian adolescents; and 2) the suboptimal quality of asthma management. The specific aims were 1) to conduct asthma surveillance in adolescents by measuring prevalence and severity following ISAAC Phase III methods; and 2) to evaluate the appropriateness of asthma management in Nigeria, especially access to affordable, quality-assured essential asthma medicines, as defined by the WHO.^13^

## METHODS

### Study population and materials

#### Study design

This was a cross-sectional study, following the GAN Phase I methodology,^14^,^15^ identical to the methodology used for ISAAC Phases I and III.^16^

#### Study population

The population of interest was adolescents in randomly selected secondary schools in the five urban local government areas (LGAs) within Ibadan City (southwestern Nigeria), and their parent(s)/guardian(s).

#### Study procedure

The sampling frame was all secondary schools in the five urban LGAs in Ibadan City, the same sampling frame that was used in ISAAC in 1995. The sample size was 3,407 to allow for an attrition of 10% and reach the target number of 3,000 students.^14^ Of the 85 secondary schools in the five LGAs that enrolled for ISAAC Phases I and III, 23 were chosen for the study. All adolescents in each chosen school formed the sampling unit. No school refused participation, and no eligible pupil was excluded from the sample.

The same standardised ISAAC written core questionnaire used in Phases I and III was used in GAN Phase I, with the addition of questions on risk factor determinants and on the management of asthma. The core questions are both sensitive and specific, with predictive validity.^16^ The core questionnaire has four sections comprising questions on demographics, eight questions on wheezing and asthma, six questions on rhinitis and seven questions on eczema. However, for this report, symptoms related to asthma were the focus. The questionnaire focused on past and current wheezing episodes. ‘Current wheeze’ was defined by a positive answer to the question ‘Have you had wheezing or whistling in the chest in the past 12 months?’ Severe asthma was defined as having ≥4 attacks of wheeze, or sleep disturbance for ≥1 night per week due to wheeze, or wheeze affecting speech in the past 12 months among those with current wheeze. A positive answer to the question ‘Have you ever had asthma?’ was used to categorise ‘asthma ever’; a positive response to ‘In the past 12 months, has your chest sounded wheezy during or after exercise?’ was used to define ‘exercise wheeze’; a positive response to the question, ‘In the past 12 months, have you had a dry cough at night, apart from that associated with a cold or chest infection?’ was used to define ‘night cough’. A positive answer to the question ‘Have you used any inhaled medicines, e.g., puffers to help your breathing problems at any time in the past 12 months (when you didn’t have a cold)?’ was used to categorise ‘purchasing and usage’.

The questionnaires were administered to the students who completed them immediately at school. Demographic questions included the participant’s name, age, date of birth, school, sex and date of interview. Questionnaires were coded to ensure confidentiality. The use of the English version of the written questionnaires was considered appropriate, as English is the official language in Nigeria, and the English literacy rate among the parents or guardians in the study population of Ibadan in southwestern Nigeria is relatively high (62.6% according to a local survey; National Bureau of Statistics. The National Literacy Survey, 2010, www.nigerianstat.gov.ng).

The protocol prescribed by GAN for height and weight measurements was employed.^15^ The data and Centre Report were sent to the GAN Global Centre in Auckland, New Zealand, for initial checking. The data were then forwarded to the data centre in London, UK, for checking and analysis using research analysis software Stata v13–15 (Stata, College Station, TX, USA).^17^ When the data and Centre Report checks were finished, a final copy of each was sent back to Ibadan.

The prevalence of symptoms was calculated by dividing the number of positive responses to each question by the number of questionnaires answering at least one symptom question (i.e., the total number of respondents in the dataset). This denominator is thus consistent across all questions/symptoms and corresponds to those in the previous ISAAC publications.^3^,^4^ The definitions of all the symptom outcomes, including severe wheeze, are also consistent with previous ISAAC papers.^3^,^4^

The 10-year change in symptom prevalence was calculated by dividing the difference between the two time points by the number of decades between the mean data collection dates of those time points. We calculated the 10-year change in symptom prevalence based on the difference between the two time points (e.g., GAN Phase I and ISAAC Phase III), divided by the number of decades between the mean data collection dates of those time points. We calculated the standard error (SE) of this change to account for school-level clustering.^18^

### Accessibility to asthma medicines

Access to asthma medicines can be defined as having medicines continuously available and affordable at public or private health facilities or medicine outlets that are within 1 hours’ walk from the patient’s home (United Nations Development Group, 2003).

### Ethics and consent

Ethical approval was obtained from the University of Ibadan, University College Hospital Institutional Review Board, Ibadan, Nigeria. Permission was sought from the schools’ authority and enrolment into the study was voluntary after explanation of the intentions and purpose of the study to the students. A letter of explanation was sent home to the parents/guardians. Assent was taken from the students before administering the questionnaire. Participants could withdraw from the study without loss of benefits.

## RESULTS

Between February and July 2018, 23 secondary schools in Ibadan City were enrolled and 3,407 questionnaires for adolescents (age: 13–14 years) were administered, of which 2,914 were returned (participation rate: 85%). After data checking, 2,897 adolescents’ questionnaires were available for analyses.

### Baseline characteristics

The Table gives the baseline characteristics of respondents. Females constituted 58% of the participants, with a male to female ratio of 1:1.4. There were 1,402 13-years-olds, and 1,494 14-years-olds. The mean weight was 45.09 kg, and the mean height 1.56 m. Using the normal body mass index (BMI) of 18.5–24.9 kg/m^2^, the majority of the participants (*n* = 1,906, 65.8%) were underweight, 901 (31.1%) had normal weight, 64 (2.2%) were overweight and 20 (0.7%) were obese.

**Table:** Baseline characteristics*

Variables	(*n* = 2,897) *n* (%)
Sex	
Male	1,217 (42.0)
Female	1,680 (58.0)
Age, years	
13	1,402 (48.4)
14	1,494 (51.6)
Missing	1 (0.0)
BMI	
Underweight	1,906 (65.8)
Normal weight	901 (31.1)
Over-weight	64 (2.2)
Obese	20 (0.7)
Missing	6 (0.2)

* Mean weight: 45.09 kg; mean height: 1.56 m.

BMI = body mass index.

### Prevalence and severity of asthma symptoms

**Figure 1:**
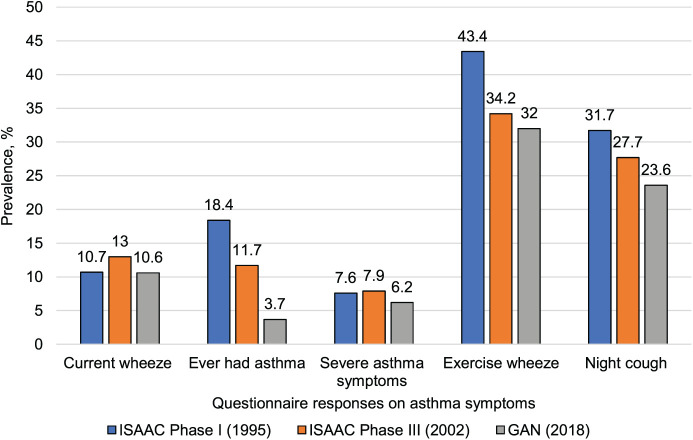
GAN questionnaire responses on asthma and asthma symptoms in previous 12 months among 13–14 year olds, Ibadan, Nigeria. ISAAC = International Study of Asthma and Allergies in Childhood; GAN = Global Asthma Network.

The 12-month prevalence of asthma symptoms for ISAAC Phases I and III, and GAN are shown in Figure 1. Comparing the data from GAN with those from ISAAC Phases I and III, there is an absolute prevalence fall across all the symptoms. Supplementary Table S1 shows the trends from ISAAC Phase III to GAN Phase I, the 12-month prevalence of asthma symptoms, the average change per decade and standard errors (SE) of the changes. The period between ISAAC Phase III and GAN Phase I comprised 16.7 years (Supplementary Table S1). For current wheeze, there was an absolute prevalence fall per decade from ISAAC Phase III to GAN Phase I of –1.4% (SE: –1). The pattern of changes in the prevalence of reported asthma ever, severe asthma symptoms and night cough was similar to changes in current wheeze, with a decrease of ≥1 SE, except for exercise wheeze (–0.7 SE). Supplementary Table S2 shows changes over the 23-year period between ISAAC Phase I and GAN Phase I. For current wheeze, there was a 0% (SE: –0.1) absolute prevalence fall per decade from ISAAC Phase I to GAN Phase I. There was a fall in the absolute prevalence of reported asthma ever, severe asthma symptoms and night cough, which were different from changes in current wheeze, and with a decrease of ≥1 SE, except for severe asthma symptoms (–0.2 SE).

### Access to asthma medicines

**Figure 2:**
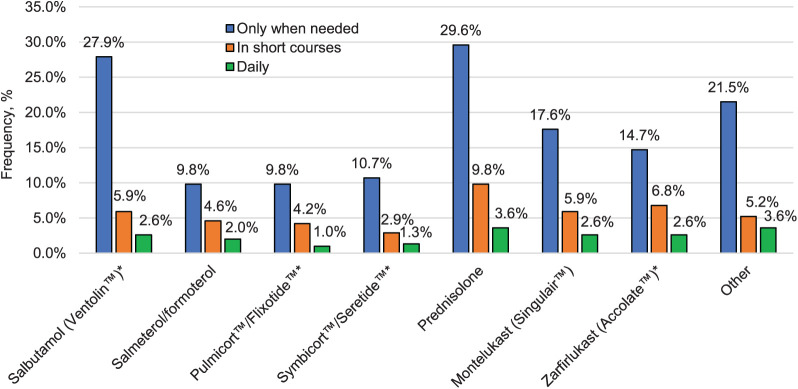
Self-reported use of asthma medications. *Ventolin™ (Glaxo SmithKline, Brentford, UK); Pulmicort™ (AstraZeneca, Cambridge, UK) = budésonide; Flixotide™ (Glaxo SmithKline) = fluticasone propionate; Symbicort™ (AstraZeneca) = budesonide/formoterol; Seretide™ (Glaxo SmithKline) = fluticasone/salmeterol; Accolate™ (AstraZeneca).

Respectively 36% and 43% of symptomatic adolescents purchased and used salbutamol and prednisolone. Less than one-fifth used leukotriene receptor antagonists such as zarfirlukast, (14.7%) and montelukast (17.6%), whereas, only about 10% had accessibility to ICS and ICS-LABA (Figure 2).

## DISCUSSION

Using methods identical to those used in the ISAAC study, the initial phase of the GAN study has yielded the first comparable assessments of the prevalence and severity of asthma symptoms among adolescent school children in almost three decades. Ibadan, Nigeria was one of the 27 centres in 14 countries participating in GAN Phase I.^10^–^12^ This study from Nigeria is part of that parent study, and this paper expands on information from Ibadan.

Between ISAAC Phase III and GAN Phase I, there was an absolute prevalence fall per decade in current wheeze, reported asthma ever, severe asthma symptoms, night cough and exercise wheeze. Over the 23 years between ISAAC Phase I and GAN Phase I, there was no change in trends for current wheeze. However, there was a fall ≥1 SE in absolute prevalence per decade of reported asthma ever, severe asthma symptoms and night cough, and a decrease of ≥–0.2 SE, except for severe asthma symptoms. The progressive fall in reported asthma ever of a much greater magnitude of any change in current wheeze suggests that there may be a progressive under-recognition of asthma by parents or health professionals or both, or that there is an increasing stigma to the use of the term ‘asthma’.

This recent decrease in prevalence of reported current wheeze is consistent with those in Western Europe, Oceania, Eastern Mediterranean and Australia.^3^ In a UK study, there was a decrease in reported wheeze in 12–14-year-olds in the 7-year period between 2002 and 1995.^19^

Obesity is linked with wheeze;^20^ however, the nutritional status of the study population suggests that only a very small minority (0.7%) were obese, within the national average of 0.0–2.8% for adolescents.^21^ Hence, the prevalence of obesity cannot explain the decrease in the prevalence of current wheeze.

Our centre participated in ISAAC Phases I (1995) and III (2001/2) and a significant rise was found in the prevalence of current wheeze over the 6-year period, from 10.7% to 13.0%.^9^ A decrease in symptom prevalence after a period of increase as documented in GAN Phase I may be due to a decrease in the intensity of an aggravating environmental factor or an increase in a protective environmental/management factor. For example, the ingestion of antioxidants may be associated with fewer asthma symptoms.^22^,^23^

The reduction in severity in this study could not be attributed to improved medical care. In Nigeria, there are very limited availability and affordability of recommended asthma medicines – ICS and inhaled salbutamol^24^ – and these are neither available nor affordable. We do not have data to estimate access of asthma medicines among adolescents, as only data on self-reported use of asthma drugs were analysed. Hence, only a small fraction of symptomatic adolescents used a reliever medication such as salbutamol, and less than half of them used corticosteroids like prednisolone. The effective control of asthma continues to pose a significant difficulty in Nigeria.^8^ This challenge is closely intertwined with factors such as poverty, poor infrastructure, a weak health system, limited access to essential asthma medications and inadequate communication between healthcare providers and patients. This study emphasises the ongoing lack of access to essential asthma medications among a significant number of symptomatic adolescents in Ibadan, Nigeria. The findings underscore the urgent need for policy makers to develop effective strategies that will enhance accessibility to healthcare for this vulnerable population.^6^,^7^

This study draws its strength from the fact that it made use of a standardised written questionnaire that has been validated in a large population-based survey which shows a substantial agreement between self-reported asthma symptoms and clinical diagnosis of asthma in adolescents.^25^ Furthermore, the study measured the trends in asthma prevalence among adolescents in Ibadan, following from the last survey conducted 16 years ago using a similar population within the Ibadan metropolis. In addition, the study assessed severity, risk factors and access to good medical care. One limitation of this study is recall bias that is a characteristic of epidemiological studies, as participants may not remember past symptoms accurately, as well as changes in the individual’s understanding of the disease.

## CONCLUSIONS

Comparatively, the prevalence of present wheezing and the intensity of asthma symptoms have notably diminished when contrasted with surveillance data from 2001-2002. However, this decrease is only evident in the severity of asthma symptoms observed in 1995, albeit still at a substantial level. While the cause of this reduction remains unexplained, there remains a clear need to ensure availability of affordable quality-assured essential asthma medications.^26^–^28^ There is an urgent need for policy makers to develop effective strategies that will enhance accessibility to affordable healthcare for this vulnerable population.
