# Supplementary material for: Decreased prevalence and severity of asthma symptoms among adolescents in Ibadan, Nigeria, 1995–2018
Source: Int J Tuberc Lung Dis. 2023 Dec 1;27(12):925–31. doi: 10.5588/ijtld.23.0138 (PMC10719893; doi:10.5588/ijtld.23.0138)
Supplement: Supplementary file 1 [file iutld_ijtld_23.0138_supplementarydata1.pdf]

http://dx.doi.org/10.5588/ijtld.23.0138

SUPPLEMENTARY DATA

Decreased prevalence and severity of asthma symptoms among adolescents in Ibadan, Nigeria, 1995–2018

Supplementary Table S1 13-14-year age group ISAAC Phase III to GAN Phase I, date of data collection, numbers of adolescents, response rate, 12-month prevalence of asthma symptoms, average change per decade and SE of the change

GAN* response rate	Mean data collection date for GAN	Years between phases	Number of individuals	Wheeze in past 12 months			Ever had asthma			Severe asthma symptoms in past 12 months			Exercise wheeze in past 12 months			Night cough in past 12 months		
				Prevalence	Absolute change per decade	Number of SEs change	Prevalence	Absolute change per decade	Number of SEs change	Prevalence	Absolute change per decade	Number of SEs change	Prevalence	Absolute change per decade	Number of SEs change	Prevalence	Absolute change per decade	Number of SEs change
		ISAAC III to GAN	GAN	GAN %	ISAAC III to GAN %	ISAAC III to GAN	GAN %	ISAAC III to GAN %	ISAAC III to GAN	GAN %	ISAAC III to GAN %	ISAAC III to GAN	GAN %	ISAAC III to GAN %	ISAAC III to GAN	GAN %	ISAAC III to GAN %	ISAAC III to GAN
85·0%	May 2018	16·7	2,897	10·6	-1·4	-1·0	3·7	-4·8	-5·3	6·2	-1·3	-1·2	32·0	-1·3	-0·7	23·6	-2·5	-1·8

*GAN Phase I

Supplementary Table S2 13-14-year age group ISAAC Phase I to GAN Phase I, date of data collection, numbers of adolescents, response rate, 12-month prevalence of asthma symptoms, average change per decade and SE of the change

GAN* response	Mean data	Years between	Number of	Wheeze in past 12 months	Ever had asthma	Severe asthma symptoms in past 12 months	Exercise wheeze in past 12 months	Night cough in past 12 months

				Prevalence	Absolute change per decade	Number of SEs change	Prevalence	Absolute change per decade	Number of SEs change	Prevalence	Absolute change per decade	Number of SEs change	Prevalence	Absolute change per decade	Number of SEs change	Prevalence	Absolute change per decade	Number of SEs change
		ISAAC I to GAN	GAN	GAN %	ISAAC I to GAN %	ISAAC I to GAN	GAN %	ISAAC I to GAN %	ISAAC I to GAN	GAN %	ISAAC I to GAN %	ISAAC I to GAN	GAN %	ISAAC I to GAN %	ISAAC I to GAN	GAN %	ISAAC I to GAN %	ISAAC I to GAN
85·0 %	May 2018	23·1	2,897	10·6	0·0	-0·1	3·7	-6·3	-3·8	6·2	-0·1	-0·2	32·0	-4·9	-2·2	23·6	-3·5	-2·9

*GAN Phase I
